# Targeting pyroptosis in breast cancer: biological functions and therapeutic potentials on It

**DOI:** 10.1038/s41420-023-01370-9

**Published:** 2023-02-23

**Authors:** Cong Chen, Qianwei Ye, Linbo Wang, Jichun Zhou, Aizhai Xiang, Xia Lin, Jufeng Guo, Shufang Hu, Tao Rui, Jian Liu

**Affiliations:** 1 Department of Breast Surgery, Affiliated Hangzhou First People’s Hospital, Zhejiang University School of Medicine, Hangzhou, China; 2 Department of Surgical Oncology, Sir Run Run Shaw Hospital, Zhejiang University School of Medicine, Hangzhou, China

**Keywords:** Breast cancer, Necroptosis

## Abstract

Pyroptosis is a lytic and inflammatory type of programmed cell death that is mediated by Gasdermin proteins (GSDMs). Attractively, recent evidence indicates that pyroptosis involves in the development of tumors and can serve as a new strategy for cancer treatment. Here, we present a basic knowledge of pyroptosis, and an overview of the expression patterns and roles of GSDMs in breast cancer. In addition, we further summarize the available evidence of pyroptosis in breast cancer progression and give insight into the clinical potential of applying pyroptosis in anticancer strategies for breast cancer. This review will deepen our understanding of the relationship between pyroptosis and breast cancer, and provide a novel potential therapeutic avenue for breast cancer.

## Facts

- Pyroptosis is an inflammatory type of programmed cell death that is mediated by Gasdermin proteins (GSDMs).
- GSDMs are abnormally expressed in breast cancer and are involved in cancer progression.
- Pyroptosis is associated with inflammatory cytokine production, which exerts effects on cancer progression and tumor microenvironment.
- Pyroptosis can be triggered by chemotherapy, radiotherapy, nanomaterials, and several small-molecule chemicals or medications.
- Triggering pyroptosis combined with immunotherapy provides a novel therapeutic potential for breast cancer treatment.

## Open questions

- Does the occurrence of pyroptosis increase or decrease the risk of breast cancer?
- What is the exact mechanism for pyroptosis to activate the immune response?
- Are there additional pyroptosis inducers and regulatory genes that remain to be discovered?
- How to reduce the side effects of pyroptosis inducers when applied clinically?

## Introduction

Breast cancer is the leading cause of cancer morbidity and mortality in women around the world [1]. Despite recent advances in breast cancer treatment, there are still a considerable number of patients who acquire drug resistance after systemic treatments such as endocrine therapy, chemotherapy, and targeted therapy, which presents a dilemma for further therapeutic intervention [2, 3]. With the in-depth research on the mechanism of tumor drug resistance, resistance to programmed cell death is one of the recognized mechanisms of tumor drug resistance [4]. Therefore, the induction of programmed cell death (PCD) in tumor cells is expected to be a significant advance in reversing drug resistance and is predicted to serve as the foundation of translational medicine in the future [5–7].

As a form of pro-inflammatory programmed cell death, pyroptosis differs from other types of programmed cell death, such as apoptosis, autophagy, and ferroptosis in terms of its morphology, biochemistry, and molecular process. In the 1990s, researchers revealed for the first time that *Shigella flexneri* and *Salmonella typhimurium* could trigger the lytic death of macrophages [8, 9]. Subsequent studies found that this type of cell death is dependent on caspase-1 activation, which is different from the traditional form of apoptosis that depends on the activation of caspase-3, and named it pyroptosis for the first time [10, 11]. Even though the fact that the concept of pyroptosis was introduced relatively early in the research process, it is challenging to make a breakthrough in the study of its exact occurrence process and molecular mechanism, resulting in relatively slow progress in the initial stages of pyroptosis research. Until recently, researchers discovered that the Gasdermin family proteins are the key execution molecules of pyroptosis [12–15], which makes the study of pyroptosis an attractive topic for cancer research.

Emerging evidence indicates that pyroptotic cell death leads to tumor growth suppression and targeting pyroptosis might be a promising anticancer strategy. Recent findings of new approaches to trigger pyroptosis in breast cancer cells and further identified the function of pyroptosis-related genes in breast cancer progression serve as a foundation for developing strategies for targeting pyroptosis in breast cancer treatment. In this review, we first present a current overview of the expression patterns and roles of pyroptosis-related molecules in breast cancer and discuss the potential impact of pyroptosis on the development and treatment of breast cancer.

## Mechanisms of pyroptosis

### Canonical pathway

The canonical pathway, which is dependent on caspase-1 activation, was the first pyroptosis pathway to be identified. When cells respond to various pathogen-associated molecular patterns (PAMPs) and damage-associated molecular patterns (DAMPs), cytosolic pattern recognition receptors (PRRs) are activated, thereby stimulating cells to form corresponding inflammasomes (NLRP1, NLRP3, NLRC4, AIM2, and PYRIN inflammasomes) [16, 17]. In addition, the inflammasome is responsible for the activation of the caspase-1 protein by recruiting an Apoptosis-associated speck-like protein containing a CARD (ASC) and procaspase-1 protein. On the one hand, the activated caspase-1 protein has the ability to cleave the GSDMD protein, release an N-terminal domain of GSDMD (N-GSDMD), and initiate pyroptosis in cells [12, 18]. On the other hand, it can cleave the precursors of IL-1β/IL-18 into mature IL-1β/IL-18 cytokines, which are then secreted outside the cells to further amplify the process of inflammatory signals and cell pyroptosis [19].

### Non-canonical pathway

The non-canonical pyroptosis pathway is mediated by the caspase proteins caspase-4/5/11, with caspase-4/5 being present in humans and caspase-11 being present in mice [20]. Bacterial lipopolysaccharide (LPS) can directly bind and activate caspase-4/5/11 and further cleave GSDMD into N-GSDMD, thereby initiating the pyroptosis program [13, 21, 22]. Unlike caspase-1, caspase-4/5/11 is incapable of cleaving pro-IL-1β/pro-IL-18. However, the non-canonical pyroptosis process can also activate the NLPR3/caspase-1 pathway, which ultimately results in the maturation and secretion of IL-1β/IL-18 [23]. Besides, N-GSDMD cleaved by caspase-4/5/11 leads to a drop in intracellular potassium levels, which could activate NLRP3 inflammasome as well [24]. In addition, cleaved caspase-11 could activate the pannexin-1 channel to induce ATP release, which in turn activates the purinergic P2X7 receptor to mediate pyroptosis [25].

### Other pathways

It was reported that several chemotherapeutic agents can induce pyroptosis in tumor cells via the caspase-3/GSDME pathway [26]. In addition, caspase-8 in tumor cells can be activated by TNF-α to cleave GSDMC [27], while in murine macrophage, activation of caspase-8 in the context of TAK1 inhibition results in cleavage of both GSDMD and GSDME [28].

**Fig. 1:**
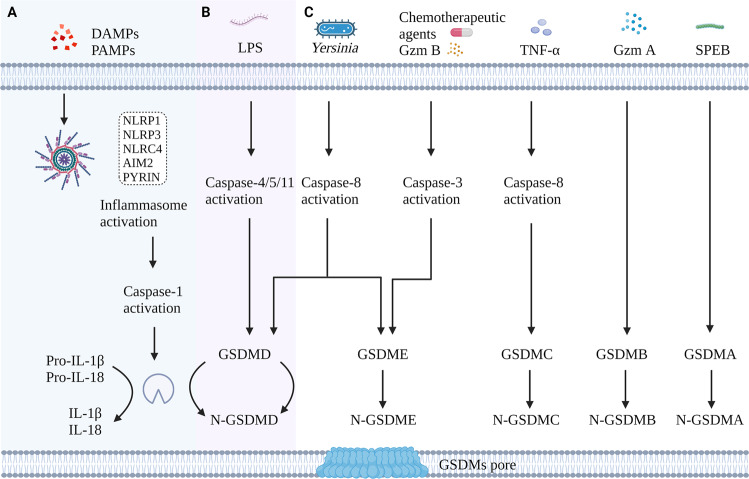
Molecular mechanism of pyroptosis. **A** Canonical pathway. In the canonical pathway, pathogens lead to inflammasome formation and activation of caspase-1, which subsequently cleaves GSDMD and promotes IL-1β/IL-18 maturation. GSDMD-N-terminal domain forms pores in the plasma membrane, further resulting in cell lysis and IL-1β/IL-18 release. **B** In the noncanonical pathway, caspase-4/5/11 is activated by cytosolic LPS, triggering pyroptosis by cleaving GSDMD. **C** *Yersinia*, TNF-αsignaling, Gzm B, and chemotherapeutic drugs involve in the activation of caspase-8 and caspase-3, which induce pyroptosis through cleavage of GSDMC, GSDMD, or GSDME. In addition, Gzm A, Gzm B, and SPEB also cause pyroptosis by directly cleaving GSDMB, GSDME, and GSDMA, respectively.

Interestingly, Granzyme A (Gzm A), which is secreted by cytotoxic T lymphocytes and natural killer cells, enters target cells with the assistance of perforin and could directly cleave GSDMB, resulting in cell pyroptosis [29]. Moreover, Granzyme B (Gzm B) can cleave GSDME in an indirect manner by activating caspase-3, or it can also directly cleave GSDME to initiate pyroptosis [30], which provides a new therapeutic strategy for future tumor immunotherapy. Recently, through a genome-wide CRISPR-Cas9 system, Deng et al. successfully identified streptococcal pyrogenic exotoxin B (SPEB) that can cleave GSDMA and lead to pyroptosis [31], which further enriches and extends the understanding of pyroptosis. The main pathways involved in pyroptosis are summarized and presented in Fig. 1

## Gsdms in breast cancer

The GSDM superfamily contains six genes (*GSDMA* to *E* and *DFNB59*) in humans [32]. Except for the PJVK protein, other GSDMs proteins feature an N-terminal domain (with cell membrane hole punching activity), a C-terminal domain (with inhibitory activity), and a hinge region structure connected in the middle [33]. Given that the GSDMs execute the ultimate function in the process of pyroptosis, scientists redefine pyroptosis as a form of Gasdermin-mediated programmed cell death [14]. Therefore, we provide an overview of GSDMs in breast cancer, with a focus on their expression profiles, regulation mechanism, and biological characteristics involved in cancer biology (Table 1).

**Table 1:** The expression and function of GSDM family members in breast cancer.

Human gene	Expression pattern	Regulation mechanism	Biological function	Prognosis	Refs
GSDMA	Down-regulated	DNA methylation	Uncertain	Uncertain	[36, 37]
GSDMB	Up-regulated	Interact with Hsp90	Promotes invasion and metastasis; Resistance to trastuzumab	Negative	[39, 40]
GSDMC	Up-regulated	nPD-L1/p-Y705-Stat3; LINC00511/hsa-miR-573	Uncertain	Uncertain	[27, 46, 128]
GSDMD	Down-regulated	Uncertain	Tumor suppressor	Positive	[51]
GSDME	Down-regulated	DNA methylation; Regulated by CDK7-P53; Upregulated by p63γ	Tumor suppressor	Methylation status as a biomarker	[57, 58, 60, 62]

### GSDMA

Humans contain a single gene for *GSDMA*, while mice have three homologs of *Gsdmas* (*Gsdma1–3*) [33, 34]. Human *GSDMA* is primarily detected in normal epithelial cells but is generally depleted in cancer cells [35]. In 2000, Saeki et al. first detected the mRNA expression of *GSDMA* in eight breast cancer cell lines and observed no transcript of *GSDMA* in any of the cell lines, even with the amplification of the *GSDMA* gene [36]. However, treatment with 5-aza-2’-deoxycytidine (5-aza-dC), a DNA methyltransferase inhibitor, could upregulate the expression of *GSDMA* but not *GSDMB* in the MCF7 cell [37], indicating that *GSDMA* expression is mediated by DNA methylation in breast cancer. Since there have been only a few studies on GSDMA in breast cancer, further studies are needed for a deeper understanding of the role of GSDMA in breast cancer.

### GSDMB

Although *GSDMA* and *GSDMB* are located at the same human chromosomal region (17q21) [38], unlike *GSDMA*, *GSDMB* is markedly over-expressed in breast cancer and acts as an oncogene [39, 40]. Interestingly, research had shown that there are four isoforms of GSDMB and different isoforms play different roles in breast cancer. In 2014, Hergueta et al. compared four variants of GSDMB level in breast cancer samples as well as normal mammary tissue and reported that only isoform 2 (GSDMB-2) was significantly up-regulated in breast cancer. Furthermore, they also documented that both GSDMB-1 and GSDMB-2 could enhance breast cancer cell metastasis in a specific signaling pathway [39]. These data indicated different splice variants of GSDMB might play a complex role in breast cancer progression and the potential mechanism has not been fully identified.

When referred to different molecular types of breast cancer, *GSDMB* is found to be intensively amplified in HER2-positive breast cancer and is related to poor prognosis. Moreover, over-expressed *GSDMB* contributes to resistance to chemotherapy and anti-HER2 therapies in HER2-positive breast cancer patients [40]. Based on the above results, researchers developed a new biocompatible nanocarrier, which works as a vehicle for intracellular delivery of an anti-GSDMB antibody into HER2 breast cancer cells. Intriguingly, targeted anti-GSDMB nanotherapy effectively restricted HER2 breast cancer cell aggressiveness and specifically increased sensitivity to trastuzumab [41]. Therefore, targeting GSDMB might serve as a promising strategy against HER2-positive breast cancer in the future.

### GSDMC

The aberrant over-expression of *GSDMC* was initially identified in metastatic melanoma cells in mice [42], suggesting that GSDMC could promote tumor progression. However, several studies had revealed that the expression profile and biological function of *GSDMC* in different tumor tissues are inconsistent [43–45]. In breast cancer, Xu et al. compared the expression level of *GSDMC* in breast cancer cells and normal breast cells (MCF10A) and proposed that *GSDMC* was markedly up-regulated in breast cancer cells [46]. Recently, Hou et al. reported that hypoxia stress and certain chemotherapeutic drugs could activate *GSDMC* expression. Mechanistically, p-Stat3 could physically bind with nuclear-translocated PD-L1 and its complexation interacts with the site of the *GSDMC* promoter, enhancing *GSDMC* expression transcriptionally in breast cancer cells [27]. Nevertheless, the specific functions of GSDMC in breast progression are still unclear.

### GSDMD

Human *GSDMD* is widely expressed in epithelial cells, immune cells, and certain cancers [45, 47–50]. A previous study conducted immunohistochemical analysis on 108 cases of breast cancer tissues and 23 cases of para-cancerous normal specimens [51]. The expression level of GSDMD in breast cancers was significantly lower than that in neighboring normal tissues. In addition, breast cancer patients with high expression of GSDMD have lower tumor clinical stage and histological grade. Likewise, the higher expression of GSDMD was related to better prognosis in breast cancer patients. These results imply that GSDMD acts as a tumor suppressor in breast cancer progression. However, the underlying mechanism remains obscure. In terms of triple-negative breast cancers, Yan et al. detected that the expression of GSDMD was remarkably upregulated after cisplatin-based neoadjuvant chemotherapy [52]. This finding might provide a new strategy for overcoming cisplatin-resistant breast cancers.

### GSDME

As reported, *GSDME* expression was frequently epigenetic silenced by methylation in several cancers [53–56], including breast cancers. In 2008, Kim et al. identified that the mRNA level of *GSDME* was significantly down-regulated in primary breast cancers compared to normal tissues [57]. However, 5-aza-dC could trigger *GSDME* expression at the transcription level in breast cancer cells, and a quantitative DNA methylation assay further proved the promoter hypermethylation of *GSDME* [57, 58]. Interestingly, the expression level of *GSDME* was markedly higher in the ER-negative compared with ER-positive breast cancers [59, 60]. As mentioned above, although *GSDME* expression is regulated by methylation, the relationship between ER status and *GSDME* methylation is still ambiguous and requires further studies [57, 60, 61].

Similarly, another study also analyzed the *GSDME* promoter methylation in a larger number of breast cancer samples and proposed that *GSDME* methylation was a potential biomarker for breast cancer diagnosis [61]. Moreover, based on the public database, researchers further investigated all 22 CpGs in the *GSDME* gene and combined two CpGs (one in the gene body, another in the gene promoter) to build a strong predictive model for detecting breast cancers [60]. In addition, survival analysis revealed that *GSDME* gene body methylation was significantly associated with patient prognosis [60]. Taken together, these findings indicate that *GSDME* methylation presents a suitable biomarker for breast cancer detection and prognosis.

In terms of the biological function of GSDME, Kim et al. conducted cell proliferation and invasion assays in vitro and revealed that GSDME acts as a tumor suppressor [57]. Consistently, Wang et al. also identified that CDK7 inhibition induced the expression of GSDME in a p53-dependent pathway, thus inhibiting breast cancer cell growth and promoting cell death [62].

## Pyroptosis in breast cancer progression

PCD plays multifunctional roles in breast cancer biological processes [63–65]. Relevant studies had verified that dysregulation of autophagy is associated with breast cancer pathogenesis and metastasis [66, 67]. Ferroptosis, which is characterized by intracellular iron accumulation and lipid peroxidation, has implications for the progression of breast cancers as well [68]. In terms of pyroptosis, emerging evidence also shows that pyroptosis participates in the physiological and pathological processes of breast cancer. As stated above, although pyroptosis-related genes could affect breast cancer progression, we here focus on the impact of pyroptosis outcomes in the development of breast cancer.

### Proliferation and invasion

Activation of tumor cell pyroptosis leads to lytic death of tumor cells, which directly inhibits breast cancer growth and proliferation [69, 70]. Therefore, inducing pyroptosis might be a novel therapeutic strategy for breast cancer. However, as pro-inflammatory programmed cell death, pyroptosis leads to the rupture of the cell membrane and the release of some intracellular cytokines, such as IL-1β and IL-18 [15]. Notably, the secreted IL-1β/IL-18 activates downstream signaling pathways by binding to their respective receptors (IL1R1, IL-18Rα, and IL-18Rβ) on tumor cells, therapy performing multiple functions in tumor progression.

It was reported that an increased level of IL-1β in serum was identified in breast cancer patients and related to an advanced stage and poor prognosis [71–73]. Meanwhile, increasing evidence had revealed that IL-1β promotes the proliferation and metastasis of breast cancer cells in vitro and in vivo [73–80]. Jang et al. revealed that IL-1β up-regulated KI-67 expression and accelerated cell cycle in breast cancer cell lines [73]. More specifically, IL-1β could directly promote epithelial–mesenchymal transition [76, 81], stimulate tumor cell adhesion to endothelial cells [82], activate matrix metalloproteinase (MMP) secretion [83], and mediate related-malignancy signaling pathways [84, 85], leading to pathological progression of breast cancers. Although mounting studies confirmed the role of IL-1β in promoting tumor progression, the opposite function of IL-1β has also been proposed. Voloshin et al. demonstrated that recombinant IL-1β does not directly affect the invasive properties of breast cancer cells in vitro [86]. At the same time, a recent finding addressed that IL-1β maintains metastasis-initiating cells (MICs) in a high mesenchymal state, which facilitates MICs invasion but prevents colonization [87], implicating that IL-1β functions both pro- and anti-tumor effects in breast cancer metastasis.

Besides, an increased serum IL-18 level was observed in the advanced-stage of breast cancer patients as well [88, 89], which was consistent with IL-1β. Furthermore, there is evidence that IL-18 promotes breast cancer cell invasion and migration [90] and is associated with the suppression of claudin-12 expression and activation of the p38–MAPK pathway [91].

Hence, pyroptotic cell death in breast cancer cells results in lytic cell death on the one hand, whereas cytokines generated during pyroptosis can also confer beneficial effects on tumorigenesis and cancer progression.

### Tumor microenvironment (TME)

TME involves primary tumor cells, various stromal and immune cells, extracellular matrix, cytokines, etc. [92]. There is emerging interest in an improved understanding of pyroptosis in dynamically regulating TME, thus modulating tumor progression and treatment applications [93–95].

Accumulating evidence indicates that pyroptosis of tumor cells can result in the reorganization of the immune microenvironment and activate tumor immunity. Zhang et al. demonstrated that Granzyme B could induce pyroptosis in GSDME-positive breast cancer cells, which, in turn, improves tumor-associated macrophage phagocytosis and tumor-infiltrating lymphocytes (CD8+ T and NK cells) counts and activities [30]. As previously stated, *GSDME* was generally epigenetic silenced by methylation, whereas decitabine increased *GSDME* expression by blocking DNA methylation. Zhao et al. constructed a biomimetic nanoparticle (BNP) loaded with indocyanine green (ICG) and decitabine, which trigger breast cancer cell pyroptosis by stimulating caspase-3 cleavage to GSDME. In this experiment, tumor pyroptosis increases the concentration of inflammatory factors (IL-6 and TNF-α) in mouse serum, as well as dendritic cell maturation and CD4+ and CD8+ cells infiltration in the TME [96]. Intriguingly, an exciting result by Wang et al. revealed that only a small percentage of tumor cells undergo pyroptosis, but the entire 4T1 mammary tumor graft is eradicated in vivo [97]. Further immune-cell-subtype analysis conducted by single-cell RNA sequencing showed that tumor cell pyroptosis significantly boosted the populations of CD4+, CD8+, NK, and M1 macrophage cells in tumor immune microenvironment, thus leading to a powerful immunogenic response in cancer. As a result, the recruited cytotoxic lymphocytes could further promote tumor cell pyroptosis by releasing granzymes, thereby developing a positive feedback system [29, 30].

Moreover, inflammatory cytokines and various DAMPs released during tumor pyroptosis are also key players in TME. Infiltration of myeloid-derived suppressor cells (MDSCs), tumor-associated macrophages (TAMs), tumor-associated neutrophils (TANs), T helper IL-17-producing cells (Th17s), T-regulatory cells (Tregs), and CAFs were reported as immunosuppression [98]. Several studies revealed that IL-1β exerts dual effects in TME by regulating different immune cells. On the one hand, IL-1β-deficient was associated with the decreased infiltration of MDSCs [99] and increased CD8+ cytotoxic T cells [100], thus activating tumor immunity in breast cancer. On the other hand, targeting IL-1β could also promote TAM polarization toward the M2 phenotype, which results in breast cancer metastasis [86]. Like IL-1β, IL-18 has both pro and antitumorigenic effects by regulating the TME in breast cancer as well. Park et al. demonstrated that tumor-derived IL-18 increased immunosuppressive CD56^dim^ CD16^dim/−^ NK cell fraction and induced PD-1 expression in these NK cells, leading to a bad prognosis in TNBC patients [101]. Nevertheless, IL-18 also promotes IFN-γ production in Th1 cells and NK cells, thus enhancing the anti-tumor ability of CD8+ cells [102].

Along with inflammatory cytokines, several DAMPs are also released during the pyroptosis of cancer cells. The high mobility group box 1 protein (HMGB1) acts as a DNA-binding protein and physiologically locates it in the nucleus. Once released during the pyroptosis, HMGB1 binds to toll-like receptor (TLR) 2/4 on the surface of immune cells, which activates transcription factors NF-κB and triggers an innate immune response by releasing TNF-α and IL-6 [103]. In addition to TLR 2/4 receptors, HMGB1 could also activate the NF-κβ and ERK1/2 pathway by interacting with the receptor for advanced glycation endproducts (RAGE) [104]. Other DAMPs, such as ATP and heat shock proteins (HSPs), bind to the corresponding receptors on the surface of antigen-presenting cells (APCs), thereby activating the adaptive immune response [105, 106].

Taken together, pyroptosis of breast cancer cells exerts a crucial role in modulating the TME and exhibits both anti-tumor and pro-tumor immunity. Therefore, in the future, deeper insights into the effects of pyroptosis in regulating TME should be delivered in greater detail.

## Pyroptosis in breast cancer therapy

### Chemotherapy

Chemotherapy plays an important role in the systemic treatment of breast cancer. According to the results of the Early Breast Cancer Trialists’ Collaborative Group (EBCTCG) meta-analysis, systemic adjuvant chemotherapy reduces the 10-year mortality of breast cancer patients by one-third [107]. Recently, several published studies have shown that conventional chemotherapeutic drugs can induce pyroptosis in breast cancer.

Yan et al. uncovered that cisplatin induces pyroptosis in TNBC in vitro and in vivo through NLRP3/caspase-1/GSDMD pyroptosis pathway [52]. Hou et al. treated TNBC cell line MDAMB231 with various chemotherapy drugs and discovered that pyroptosis was only observed with types of antibiotics (daunorubicin, doxorubicin, epirubicin, and actinomycin D) by activating nPD-L1/GSDMC pathway [27]. Similarly, Zhang et al. applied paclitaxel, cisplatin, doxorubicin, cyclophosphamide, and 5‐fluorouracil to MDAMB231 and T47D cell lines and observed that doxorubicin triggers typical pyroptotic morphology in both cell lines. Furthermore, doxorubicin treatments induced ROS accumulation, which stimulated the phosphorylation of JNK, and induces pyroptosis via the caspase3/GSDME pathway [108]. However, as mentioned above, the *GSDME* is regulated by methylation in breast cancer, which heavily limits the induction of pyroptosis by chemotherapeutic drugs through GSDME protein cleavage. Fan et al. develop a strategy of combining decitabine (DAC) with chemotherapeutic nanocarriers. As a result, DAC was performed to up-regulate the expression of *GSDME* through demethylation, thus enhancing the efficacy of chemotherapeutic agents in inducing pyroptosis [109]. Recently, Li et al. presented a carrier-free chemo-photodynamic nanoplatform (assemble cytarabine with chlorin e6) that could effectively triggering pyroptosis in breast cancer as well [110].

### Immunotherapy

Immunotherapies include immune checkpoint inhibitors (ICIs), tumor vaccines, monoclonal antibodies, and adoptive immune cell therapies. Notably, ICI is a promising therapeutic strategy for some cancer types (such as non-small cell lung cancer or melanoma) and brings hope to breast cancer, especially for TNBC. Compared to other types of breast cancer, TNBC is considered a good candidate for ICIs because of the relatively higher tumor mutational burden (TMB) and higher tumor-infiltrating lymphocytes (TILs) density [111, 112]. However, in the field of TNBC immunotherapy, the efficacy of ICIs, when used as monotherapies, is not satisfactory [113]. Therefore, improving the efficacy of immunotherapy for TNBC is expected to significantly improve the prognosis of patients and is a great issue in current cancer research.

The predictive role of pyroptosis on the response to breast cancer immunotherapy remains elusive. On the one hand, the induction of pyroptosis of lymphocytes around tumors will help immune evasion, and on the other hand, pyroptosis can also activate anti-tumor immunity. In breast cancer, hypoxia-induced upregulation of intracellular and extracellular gp96, which induces CD8+ T cell pyroptosis through the GSDMD-dependent pathway and facilitates immune evasion [114]. As mentioned above, pyroptosis of tumor cells and the release of inflammatory cytokines result in the reorganization of TME, which will help to improve the response rate of tumor immunotherapy. In a mouse breast cancer model, the researchers coupled GSDMA3 protein to nanoparticles and further delivered it into mouse tumor tissue. The results delivered that although <15% of tumors suffer from pyroptosis, a large number of inflammatory factors and activated cytotoxic T cells released during pyroptosis subsequently, thereby amplifying the anti-tumor immune response and significantly sensitizes 4T1 tumors to anti-PD1 therapy [97]. Similarly, Su et al. applied oncolytic viruses with inhibitor nanoprodrugs MPNPs to synergistically ignite GSDME-mediated pyroptosis, thus reversing an immunosuppressive tumor microenvironment and increasing the efficacy of anti-PD-1 therapy [115].

In addition to regulating tumor immunity through pyroptosis, approaches that activate anticancer immunity and pyroptosis at the same time are of increasing interest. In a mouse model of breast cancer, Zhang et al. showed that the TME of *Gsdme*^−/−^ mice was associated with fewer TILs and TAMs [30]. Therefore, overexpression of endogenous *GSDME* in breast cancer is expected to simultaneously activate pyroptosis and promote immune cell infiltration, thus effectively suppressing tumor growth. Elion et al. proved that RIG-I activation induces pyroptosis in breast cancer cells through the caspase-1/GSDMD canonical pathway. Meanwhile, RIG-I agonist activates innate immunity and increases breast TILs in TME, and the combination of RIG-I agonist with anti-PD1 therapy significantly suppressed tumor development to a greater extent than either agent alone in vivo [116]. Taken together, pyroptosis might elicit additive or synergistic effects of immunotherapy for breast cancer.

In clinical practice, the efficacy of immune checkpoint inhibitors (ICIs), when used as monotherapies, is not satisfactory [113, 117], and the oncologist tends to combine ICIs with chemotherapeutic regimens for breast cancer treatment [118]. Nonetheless, it is worthwhile to investigate which chemotherapeutic medicines are most effective when combined with ICIs. In the TONIC trial, metastatic TNBC patients were randomized to nivolumab with different methods of induction therapies, consisting of irradiation, cyclophosphamide, cisplatin, or doxorubicin [119]. The results revealed that the objective response rate (ORR) was 20% for all patients and the majority of ORR was observed in doxorubicin cohorts. Moreover, three phase-III clinical trials (KEYNOTE522 [120], IMpassion031 [121], and NeoTRIP [122]) evaluated the potential efficacy of ICIs with neoadjuvant chemotherapy regimens in early-stage TNBC patients. Unlike KEYNOTE522 and IMpassion031 trials, patients involved in the NeoTRIP trial were not administered doxorubicin or epirubicin treatment. However, in the NeoTRIP trial, the combination of atezolizumab with nab-paclitaxel and carboplatin did not significantly increase the proportion of pathological complete remission (pCR) in TNBC patients, compared to no atezolizumab patients (OR 1.18; 95% CI 0.74–1.89; *P* = 0.48) [122]. Since in vitro tests have shown that anthracyclines can cause pyroptosis in breast cancer cells [27, 108], we hypothesized if these improvements in ICIs treatment would be attributable to anthracycline-induced pyroptosis in breast cancer cells. Therefore, a thorough knowledge of the molecular mechanism and process of chemotherapeutic drugs-induced pyroptosis in breast cancer cells can assist locate more suitable therapeutic adjuvants in breast cancer immunotherapy, thereby strengthening the efficiency of ICIs for breast cancer patients.

### Other therapies

Although pyroptosis has been studied extensively in chemotherapy and immunotherapy, its effect on the endocrine treatment and radiation for breast cancer is still ambiguous. Estrogen receptor alpha (ERa), a transcription factor encoded in the *ESR1* gene, is a positive prognostic factor for endocrine therapy in breast cancer. In vitro, IL-1β induces down-regulation of ERa expression by inducing methylation of the *ESR1* promoter, thus promoting tamoxifen resistance in hormone receptor-positive breast cancer cells [123]. Pham et al. treated breast cancer cells with different three inflammasome inhibitors that suppress inflammasome activity at distinct phases [74]. According to the results, treatment with inflammasome inhibitors appeared to inhibit the proliferation of breast cancer cells in estrogen receptor-positive breast cancer cells (MCF7 and T47D), but this effect was not observed in triple-negative breast cancer cells (MDAMB231). These findings prompted researchers to further investigate the relationship between the estrogen receptor signaling pathway and the pyroptosis system in breast cancer cells.

Radiotherapy is another major treatment for breast cancer. Cao et al. represented that irradiation could induce pyroptosis in GSDME high-expressing tumor cells and could effectively promote the infiltration of CD8+ T lymphocytes [124], which provides a novel therapeutic strategy for the effects of radiotherapy. In addition, as a novel tumor ablation technique, high-frequency irreversible electroporation (H-FIRE) eliminates breast cancer cells through necrosis and pyroptosis as well as a transition in the TME from anti-inflammatory to pro-inflammatory, which stimulates a systemic anti-tumor immune response [125].

With the development of nanotechnology, there are promising application prospects for the use of nanocarriers loaded with antitumor drugs for the targeted therapy of breast cancer. Zhao et al. developed a biomimetic nanoparticle (BNP) loaded with indocyanine green and DAC. On the one hand, low-dose laser irradiation at the tumor site enhanced intracellular Ca^2+^ concentration, which triggers the release of cytochrome c and the activation of caspase-3 in breast cancer cells. On the other hand, the released DAC upregulates *GSDME* expression via suppressing DNA methylation, leading to powerful cancer cell pyroptosis [96]. Therefore, the nanoparticle is a very promising technique to show site-specific delivery of multiple drugs to result in combination therapeutic effects in breast cancer treatment [126].

Meanwhile, researchers have uncovered that several small-molecule chemicals or medications can induce pyroptosis in breast cancer cells and we summarize these pyroptosis-inducing drugs in Table 2.

**Table 2:** Targeting pyroptosis in breast cancer-currently available pharmaceutical agents.

Pyroptosis inducers	Cell lines	Pyroptosis pathways	Years
Omega-3 docosahexaenoic acid [129]	MDAMB231, 4T1	Caspase-1/GSDMD	2018
hUCMSCs [130]	MCF7	NLRP1 or caspase-4	2020
Metformin [131]	MCF7	Caspase-3/GSDME	2020
Nobiletin [132]	MCF7, BT549	Caspase-1	2021
Tetraarsenic hexoxide [69]	HS578T, MDAMB231	Caspase-3/GSDME	2021
Triclabendazole [133]	MCF7, MDAMB231	Caspase-3/GSDME	2021
Cadmium [134]	MDAMB231	Caspase-3/GSDME	2021
Dihydroartemisinin [135]	MCF7, MDAMB231	Caspase-3/GSDME	2021
Cetuximab [136]	MDAMB231, MDAMB468	GSDME	2021
Polyl:C [137]	HS578T, BT549	Caspase-3/GSDME	2021
Spatholobus suberectus Dunn [138]	MDAMB231, BT549, 4T1	GSDME	2021
Trimethylamine N-oxide [139]	66cl4	Caspase-3/GSDME	2022
3-acyl isoquinolin-1(2H)-one [140]	MCF7, MDAMB231	Caspase-3/GSDME	2022
CDK inhibitors [141]	MDAMB231, BT549, 4T1	Caspase-3/GSDME	2022
Bacterial outer membrane vesicles [142]	4T1	Caspase-1/GSDMD	2022
Hirsutinolide natural product R001 [143]	MDAMB231, MDAMB468	Caspase-1/GSDMD	2022
US-enhanced enzyodynamic tactics [144]	4T1	NLRP3/GSDMD	2022

## Conclusions and future perspectives

Pyroptosis, a newly raised category of programmed cell death that is dependent on Gasdermin family proteins, has made further progress in recent years. Notably, a comprehensive understanding of the expression patterns and activities of the Gasdermin family members will assist in identifying the likelihood of pyroptosis in breast cancer cells and provide a foundation for future clinical translational research. However, in addition to being expressed in breast cancer cells, the Gasdermin family members are also abundantly expressed in normal human tissues [32, 127]. As a result, pyroptosis-inducing drugs will inevitably cause harm to normal tissue cells and a variety of undesirable effects when used on the human body. Therefore, when employing pyroptosis in the therapy of breast cancer, how to achieve a precise and effective treatment mode will be the focus of our follow-up research.

As described above, several traditional chemotherapeutic drugs (such as cisplatin, doxorubicin, etc.) have also been shown to induce pyroptosis in breast cancer cells, which not only kills tumor cells directly but also modifies the tumor microenvironment. This procedure assists in transforming “cold” cancers that are resistant to immunotherapy into “hot” ones that are responsive to immunotherapy. Besides, promising results have been shown when combining the function of pyroptosis with ICIs in breast cancer. For this reason, pyroptosis may offer innovative solutions for breast cancer immunotherapy.

Nevertheless, compared with the issues that have been resolved, additional mysteries remain to be addressed. Does the occurrence of pyroptosis increase or decrease the risk of breast cancer? How to weigh and avoid the adverse effects of pyroptosis inducers on breast cancer patients as much as possible? Is the effectiveness of pyroptosis inducers combined with ICIs limited to TNBC, or does it hold true for other molecular subtypes of breast cancer as well? Like the combination of pyroptosis with chemotherapy, is there a possibility of achieving a synergistic impact by combining endocrine therapy and anti-HER2 therapy with pyroptosis in the treatment of breast cancer? How does pyroptosis interact with other forms of PCD (autophagy, ferroptosis, etc.), and would combining pyroptosis with these other forms of PCD increase the therapeutic efficacy of breast cancer treatment? Exist any additional medications that efficiently induce pyroptosis in breast cancer cells and will be applied clinically? Many concerns must be solved before pyroptosis was formally applied in clinical settings.

**Fig. 2:**
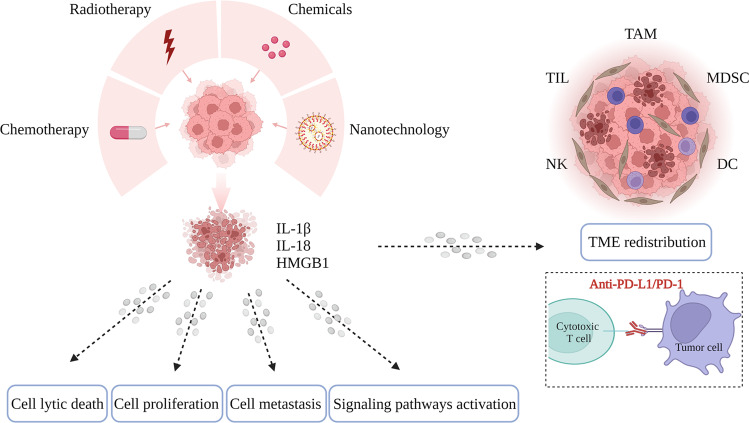
The dynamic role of pyroptosis in breast cancer tumor development and treatment. Chemotherapy, radiotherapy, and several small molecule chemicals or medications can induce pyroptosis in breast cancer cells, which results in cell lytic death, regulates cell proliferation and metastasis, and activates signaling pathways. Moreover, pyroptosis in breast cancer cells also exerts a crucial role in TME redistribution and is expected to improve the efficacy of ICIs.

Taken together, our review summarizes the expression patterns and functions of Gasdermin family proteins in breast cancer and discussed the potential influence of pyroptosis on breast cancer progression and treatment strategies (Fig. 2). With a deeper knowledge of the physiological process and molecular mechanism of pyroptosis, more and more findings will be presented in the future. These will provide new ideas and methods for the choice of treatment decisions for breast cancer patients.
